# Implementation of a neighbourhood care model in a Scottish integrated context—views from patients

**DOI:** 10.3934/publichealth.2019.2.143

**Published:** 2019-04-17

**Authors:** Calum F Leask, Andrea Gilmartin

**Affiliations:** 1 Aberdeen City Health & Social Care Partnership, Aberdeen, UK; 2 Health Intelligence Department, NHS Grampian, Aberdeen, UK

**Keywords:** Buurtzorg, community, healthcare, social care, self-management, integration

## Abstract

There is a need to test new models of integrated health and social care, particularly due to increasing financial and epidemiological pressures on services. One critical component of testing new models is the acceptability to patients. Here, the aim was to understand the acceptability of a new model of care to patients by understanding their experience of being supported by a self-managing, community-based, integrated, health and social care team. The INCA service consisted of three support workers and three nurses in two teams. These teams were self-managing and had autonomy over service operations and care delivery. Eight interviews and satisfaction questionnaires were conducted with patients. Interviews were transcribed and analysed thematically. Patients found the service highly acceptable (mean overall satisfaction of 98%), self-reporting a variety of benefits to their wellbeing. Central to this acceptability was the autonomy of staff to adjust care frequency and duration to patients' needs, in addition to describing an active engagement and partnership within their support plans. Future work should aim to ascertain the experiences of staff working in this model and whether receiving support in this way improves clinical outcomes.

## 1. Introduction

The demands on health and social care services are rising, with people living longer with multiple long-term conditions and increasingly complex needs [1]. Patients over 85 are seven times more likely to have an emergency hospital admission compared to those half their age, with bed day occupancies in this cohort growing by 76% in the last decade [2]. This demand, coupled with financial pressures, has resulted in National Health Service (NHS) spending failing to match the challenges associated with an ageing population [2]. The nature and scale of the above has resulted in a movement towards transformation of care delivery, with new, integrated models necessary to reduce pressure in acute settings by improving the capacity of community-based services. This aligns with Governmental commitments for ensuring that individuals can live longer and healthier lives in a homely setting, with health and social care integration seen as a pivotal enabler to achieving this vision [3].

When developing new community care initiatives, a critical component is to assess the acceptability to those receiving the service, allowing for a deeper understanding of to what extent its implementation meets their needs [4]. Highly acceptable interventions have been shown to increase adherence to treatment recommendations and improve clinical outcomes [5]. Therefore, it is imperative that, to develop and deliver new initiatives that are fit-for-purpose, patients' views regarding their implementation should be sought.

One model of care gaining increasing attention internationally is the Dutch model: Buurtzorg. This is characterised by nurses working in self-managing teams and advocating autonomy over all aspects of service delivery, such as care frequency, duration and working rotation [6]. Teams work closely with general practitioners and other community healthcare providers, founded on principles of relationship-based practice and empowerment, in order to improve outcomes for patients [7]. The service provided, from the patients' perspective, a higher standard of care comparative to other organisations and features consistently among the top 10 in the Netherlands for client acceptability and satisfaction [8]. Economically, Buurtzorg's average annual homecare costs per client were €6428 compared with an average for all others of €7995, thus providing a higher quality of care for less costs [9]. Despite these benefits, the principles of this model have yet to be tested within the Scottish health and social care system.

The aim of this paper is to explore the acceptability of a new neighbourhood care model by understanding the experiences of patients being supported by a self-managing, community-based, integrated, health and social care team.

## 2. Materials and methods

### 2.1. Service overview

The Integrated Neighbourhood Care Aberdeen (INCA) service was launched in February 2018 by Aberdeen City Health and Social Care Partnership (ACHSCP). As part of the Scottish Government's commitment to integrate health and social care in order to improve effectiveness and efficiencies in service provision [3], ACHSCP are responsible for delivering the integration agenda at a local level, with the INCA service being one of several piloted initiatives. The service consisted of two teams, comprised of three nurses and three support workers in each. Teams were based in two separate sites across the city—one co-located in a GP surgery in the West Locality and another based in a corporate office suite in the South Locality. The teams were self-managing and had full autonomy over service delivery, including provision of care and staff rotas. A coach was provided as a support mechanism for team-building. This contrasts with how care is traditionally delivered, whereby both nursing and social care staff assess and deliver care separately, even if they are supporting the same individual. Inclusion criteria of patients were: living in the relevant postcode area and in no previous receipt of social care / nursing need. All patients that did not meet these criteria were excluded.

### 2.2. Research design

In total, 43 patients were supported over the first four months of implementation, with individual interviews conducted with eight patients from across both sites. Demographic information collated included: age; sex; primary referral reason and referral source. Satisfaction questionnaires were also administered, assessing components including: promotion of independent living; choice of received support and perceived confidence in teams. These components were chosen to closely mirror the underpinning principles of Buurtzorg, such as self-management and enablement [6].

As the data collection fell within the remit of service evaluation, ethical approval was not necessary to acquire.

### 2.3. Protocol

Interviews were based on a semi-structured topic guide. Discussions were based on a series of exploratory questions regarding patients' experience of being supported by the INCA teams. Example questions were: *“Tell me about the support you get from the INCA team?”* and *“Have you noticed any changes to your health and wellbeing as a result of seeing the INCA team?”* Interviews lasted no more than 60 minutes and were audio recorded. Fieldnotes were also taken during discussions and used as a reference point during analysis.

### 2.4. Data analysis

Audio recordings were transcribed verbatim and analysed thematically using NVIVO Version 11 (QSR International, Doncaster). Thematic analysis is useful towards understanding patterns occurring in the data in order to improve understanding on a particular topic [10], such as the experience of being cared for by a self-managing, integrated, health and social care team. Analysis followed the six step framework previously described by Braun and Clark [11], including: 1) familiarisation with the data; 2) developing initial codes; 3) searching for themes; 4) reviewing themes; 5) theme definition and 6) write up of results. The data were analysed independently by two researchers and then findings compared and adapted if required.

## 3. Results

### 3.1. Participants

Table 1 outlines the characteristics of patients whom were interviewed in Site 1 (C) and Site 2 (P). The number and sex of interviewees was consistent across both sites, with one man and three women from each participating. The cohort were predominantly older (mean age = 83 years) and were referred onto the caseload for a variety of reasons associated with ageing.

**Table 1.** Participant demographic information.

Participant ID	Age	Sex (M/F)	Referral reason	Referral source
C1	95	F	Mobility	Social care management
C2	93	M	Mobility	Family
C3	91	F	Heart failure	General practitioner
C4	84	F	Type 2 Diabetes	Family
P1	86	F	Cancer	General practitioner
P2	85	F	Frailty	District nursing
P3	64	F	Multiple sclerosis	Hospital discharge team
P4	66	M	Lung disease	Social care management

### 3.2. Satisfaction questionnaire responses

Table 2 shows the average scores of questionnaire components across both sites. All constructs were measured using likert-scales, scored from 1 (strongly disagree) to 5 (strongly agree). The highest average scores across all components were patients feeling well-informed about their care delivery and overall satisfaction with the support they received (4.9/5). Across both sites, “increasing available choices” received the lowest average score (3.4/5).

**Table 2.** Satisfaction questionnaire scores (N = 8).

Questionnaire components	Site 1 Average Score	Site 2 Average Score	Total Average score
Prevention			
* Independent living*	4.8	4.5	4.6
* Reduce symptoms*	3.7	4.7	4.2
* Well-informed*	4.8	5	4.9
* Care well-explained*	5	4.5	4.8
Choice			
* Input of support*	4.8	4.8	4.8
* Things that matter*	4.8	4.5	4.6
* Encouraged to input*	4.8	4.8	4.8
* Increase available choices*	3.5	3.3	3.4
Overall satisfaction			
* Satisfied with support*	4.8	5	4.9
* Recommend support*	5	5	5
* Confidence in teams*	5	5	5
* Well-coordinated care*	4.5	4.75	4.6

### 3.3. Themes

Table 3 outlines the four themes that emerged from thematic analysis: 1) Service Operation; 2) Staff Qualities; 3) Acceptability & Assets; 4) Confounding Factors. Each of these themes had a number of identified sub-themes that are described below.

**Table 3.** Themes and sub-themes derived from interviews.

Theme	Sub-theme
Service Operation	Care content
	Collaboration
	Delivery mechanisms
	External support
Staff Qualities	Compassion
	High quality staff
	Respectfulness
	Supportive
Acceptability & Assets	Patient characteristics
	Patient outcomes
Confounding Factors	Care discontinuation
	Consequences of ageing

### 3.4. Service operation

**Care content:** There were numerous examples described by patients of the care that they received, unique to their individual needs. For example, the INCA teams were able to support patients with aspects of personal care: *“They come to help you with the showers and that”* (P1), as well as providing clinical care to those who required it: *“The support I need at the moment is for my ears and eyes also my foot”* (P2).

As the INCA model did not restrict patients to pre-assigned times and days of care delivery, staff had the autonomy to arrange home visits at a time that was mutually beneficial: *“Initially it was first thing in the morning to get me up and then at bedtime to get me back into bed”* (P3). Managing their own time also meant that staff could be flexible, with patients providing examples of longer or shorter periods of support as required: “*There has been one or two changes of course but there are four of them coming in here at different times*” (P2). Over time, as patients became more independent, the number of visits they received reduced accordingly: “*In five months they got me from three times a day to be independent enough to have them just coming in once in a while, just a courtesy visit*” (P4).

**Collaboration:** Patients described forming a working partnership with staff and having active input regarding what care they received, for example: “*We talk about it and I have suggested about changing my going to bed time could be a bit earlier... is an opportunity if there is something I want to say or something I need help with*” (P3). Patients also commented how the INCA team ensured their unpaid carers were involved in decisions around the support they received. This resulted in feelings of ownership in the care process and an associated open dialogue between families and staff: “*So say they came in and X [patient's daughter] phoned me then the Nurse would have a word with her and I will say ‘do you want to tell X anything?’ it gives X confidence because she'll say ‘are you sure you are alright mum, you're not just saying that?’. No, I'm not. So they know and they try to involve people, but in a nice way*” (C1).

Good communication with staff was not only seen to encourage patients to take control of their own health, but led patients to express feelings of empowerment: “*I mean I like to do little things for myself and they will leave me to do it. You know, so I said ‘don't fuss over me, if I need you I'll shout’, so they have all just rallied round. That's about all I can say really. They respect your wishes*” (C4). Patients felt that the strong alliances that they had with staff directly contributed towards the re-ablement process: “*We agreed between us they would come in twice a day and then eventually as I was recovering and getting better through physiotherapy*” (P4).

**Delivery Mechanisms**: Patients unanimously agreed that the care delivered to them was positively received. Aspects that they specifically commented on included the availability of the team: “*If I need them, I phone them and they will be down*” (P2), the stability of care provided: “*I don't feel abandoned, I feel supported*” (P3) and the overall reliability of staff: “*They are always here about the time they say they will be*” (P1).

Patients were conscious of staff having to attend others on their caseload with varying needs, signalling their appreciation for the amount of input they received: “*I am aware that there is certainly more than me around and I don't know how health conditions are for people out with the area*” (P3). One patient did remark that they felt staff had excessive paperwork to complete and was unwieldy to store within their home: “*I have got stuff lying through there, on that dresser and they are writing in that writing books, I wonder if they are writing a book about me, it's taking up a lot of space and I have to keep that space for them, will just throw it in the bin because that's what's going to happen to it*” (C2).

**External Support**: Patients described the INCA team working with their family/friends where possible to enhance external support structures that facilitated patient mobility outside their home. The assistance provided by family members in turn aided staff by contributing towards patient re-ablement: “*I cannot get out by myself but then I don't need them to take me out because my son's .... I know if I phoned them, any of them, they would be here. I know they would*” (P2). Family and friend involvement in patient care ensured that, where possible, patients continued with everyday activities and experienced social inclusion: *“I have my cousin who stays about two minutes from here and we go shopping on a Tuesday... X [friend] and I meet every week and we do it together, she is very helpful*” (P3).

Regarding their network of support, patients reported being signposted to relevant community groups, such as tea dances or Men's Shed: “*Well they asked me if I would like to go [to a community group]*” (C4). However, patients did identify barriers to attending these services, such as no wheelchair access: “*I am on two crutches and then when I do go out I need a wheelchair and getting into places, sometimes there is no access for wheelchairs*” (C3). Others did not perceive barriers to attending community assets, however felt them unnecessary given the strong family connections they had locally: “*Yes, they did [signpost], but I think meantime I've got my daughter and granddaughter”* (C1).

Recognition was given to the INCA staffs' ability to engage with other professionals to provide additional support when required by patients. For example, the team were able to quickly gain patients access to further services that they otherwise had found challenging to receive: “*If we wanted to ask about some other service or something, they might be able to put in their outlines. I had said to the clinic that I hadn't seen a Physiotherapist since I had come home and then they saw about this for me... if we are wondering about something, some other help or something, they would try and find out for you*” (P1).

### 3.5. Staff qualities

**Compassion**: Patients expressed a genuine concern from staff regarding their safety and wellbeing. For example, one individual described a recommendation from a staff member to have alarm systems installed in case of an emergency, for when team members may not be there to assist: “*They have been concerned about my safety since day one... they will not let me get myself into any dangerous situation. If they felt it was not appropriate for me to do something, they wouldn't let me do it... INCA suggested that I get a panic button... and I thought ‘brilliant!’*” (P4). These feelings extended to ensuring disability aids in the home were used correctly and safely: *“We go upstairs on my...I've got a lift, so they see that I am on my lift right and see that I am strapped in”* (C1), in addition to staff providing supervision when patients were trying new aids/equipment to facilitate their recovery: “*When I got the walker for a start, I was able to go out, somebody took me sometimes... so we just walked around the corner and back again which was very kind of them*” (P1).

**High quality**: The standard of care provided by staff was consistently acknowledged by interviewees. In particular, they commented on the uniqueness of the team members, particularly by comparison to others they may have interacted with previously: *“That's what I always say, when they made X they threw away the mould, because you don't get many like her”* (C2). Patients also appreciated being one within the geographical catchment area of the pilot site, emphasising that there were thankful for how responsible the staff were: “*I think they do a good job. We are very fortunate here to have them and they never fail to come in so that's good*” (P2).

**Respectfulness**: Patients described being treated with dignity, beyond what they would typically expect from a professional: “*I mean there's nothing that she wouldn't do for you... she takes time to have a chat with you... you can speak to them. You know they would take time and listen to you*” (C4). These qualities resulted in patients having strong feelings of trust: “*I can depend on somebody to do something about it... I needn't feel I'm alone*” (C1) and that staff were providing a person-centred service: “*The girls have been helpful, they have come in and if I want anything done then they will do it*” (C3).

**Supportive**: Although staff ensured that patients were not attempting to unsafely escalate the re-ablement process, it appeared that patients gained motivation from the team to aid their recovery, for example by discouraging sedentary behaviour: “*They were super in encouraging me to not just sit about. They got me going and encouraged me to get up to go to the bathroom and back and ... because they were encouraging me so much to get me going*” (P4).

### 3.6. Acceptability and assets

**Patient Characteristics**: Patients described a desire not to become dependent on the care that they were being provided and instead, discussed a shift towards self-managing elements of their health: *“I am trying to be self sufficient as much as possible. I do what I can”* (P2). Even though service provision was free and tailored to the individual, it was evident that patients felt retention of control and continuing to complete tasks of everyday living they were capable of doing was important: “*There are a few things that I can do myself and I keep saying to them ‘no, don't make me redundant all the time’*” (C4).

**Patient Outcomes:** Interviewees were agreed in detailing the positive impact the INCA team had on their wellbeing. For some, simply receiving a telephone call to alert them of an upcoming visit had a positive effect on self-assurance: *“Really helps my confidence as I know someone is coming and that is a big thing for me anyway knowing that someone will be along”* (P3). This model of care and support appeared to build on patient's self-efficacy, with patients more likely to attempt to do more by themselves, knowing that support was at hand: *“As long as they are here when I am showering, I have no confidence to go in the shower myself, but they sit here and if I need them I shout”* (C4). Furthermore, patients' spoke of the learning experience that existed through detailed interactions and building relationships with the staff and provided examples whereby they had made positive changes to lifestyle behaviours over time: “*I am learning more and more as the time goes by and just watching my diet more than anything else*” (P3).

For some patients who had reduced mobility and were socially isolated, the companionship that the staff provided resulted in improved mental wellbeing, such as reduced feelings of loneliness: *“I know they are coming and I am grateful for them to come in just to speak to because there is nobody else ... I like their company when they come in...I have made friends”* (P2). In addition to personal outcomes however, patients described the relationships that they formed with staff over time that went beyond simply providing care, but into friendship: “*I just used to look forward to her visits and hear about her grandchildren and she heard about mine and that was just the highlight of my day*” (C4).

### 3.7. Confounding factors

**Care discontinuation**: During the time of interviewing, challenges with staff retention resulted in care being discontinued in one site. This was a consequence of double-running the service alongside traditional care delivery in a small geographical area, meaning limited nursing input was referred into the team. This had a direct impact on patients' experience, all of whom reported disappointment in their support coming to an end: “*I'm getting them moulded into my way and you are taking them away and putting them someplace else*” (C4). There was reference from a number of patients who appreciated the low staffing numbers in each team and this was identified as a possible consequence to staff moving on: “*There is often one Carer on alone to do the whole thing. That is hard going for one person... but they are especially busy in the morning*” (P2).

**Consequences of ageing**: Despite the high-quality of support described, some patients acknowledged that simply the process of living into old age had a deleterious effect on their health: “*I could do a lot more before*” (P2). However, these feelings of ill-health did not relate to the care received, but to patients' capabilities pre-referral to the team: *“I am not managing so well now”* (P1).

## 4. Discussion

The aim of this study was to understand the experiences of being supported under a new model of care, characterised by integrated health and social care teams self-managing in the community. In order to determine whether it is feasible to implement and scale localised tests of change, it is critical to understand the acceptability of these models to those receiving the service [12]. Overall, this service appeared to be highly acceptable to patients, with overall satisfaction scoring an average of 98%.

Components within the choice element of feedback that patients strongly agreed with were their input into the support they received (average score 4.8/5), along with the team encouraging patients to have their say (average score 4.8/5). These quantitative findings are supplemented by the collaboration sub-theme that emerged from interview analysis, with patients often referring to the team-working that occurred between the two parties. This highlights the perceived benefit to patients of having equality in the relationship with those who support them. Indeed, the National Institute for Health and Care Excellence have released specific guidelines stipulating the need to ensure that patients are active participants in the care and support that they receive [13]. These guidelines are reinforced by previous evidence demonstrating that joint decision-making leads to increased adherence to treatments [14] and improve knowledge of available options [15]. Given that the Buurtzorg principles on which this model was founded emphasise the importance of placing the patient in the centre of their care needs [7], it would appear that this component of the model worked well.

One reason that may have attributed to the high patient satisfaction was the ability of the team to be agile in their care delivery. For example, patients described circumstances where their health would fluctuate and would subsequently require more or less support. The autonomy the team possessed to escalate and de-escalate frequency and duration of support contrasts to traditional models, principally in social care, whereby care provision is fixed and requires reassessment to increase [16]. This would appear to be a particularly beneficial component of delivery care in community settings, especially considering the predominantly older cohort whom received care (mean age = 83 years), a population that report large variances in their health status from day-to-day [17]. Being able to tailor care delivery to compliment the needs of patients has been attributed as one of the key components of the Buurtzorg model in improving support for frail older adults [18].

Another important principle of the model incorporated within was the mobilisation of community assets and social networks to support patients towards enablement [6]. Here, participants all provided examples of signposting they received towards other forms of support locally, such as community groups and activities. Community assets have previously been championed as offering the potential to enhance quality and longevity of life by improving coping abilities and self-esteem of individuals [19]. Despite this however, there was a reticence to attending community assets, with patients citing logistical challenges and feelings of discomfort as barriers to attend new activities. Therefore, whilst strong relationships were formed between patients and staff, further support is required to integrate individuals into sources of community assets should they desire.

## 5. Conclusion

It appears that receiving care from a self-managing, integrated, health and social care team is acceptable to patients. In particular, empowering professionals with the autonomy to adjust frequency and duration of care provision facilitated a tailored approach, therefore it is recommended that care providers implement a similar method to ensure services are person-centred. Further, considering the positive impact that informal networks and community assets can have on an individual's wellbeing, appropriate thought and subsequent resource should be given to build and maintain relationships between services and these groups. Future work should determine whether this model of care results in clinically significant improvements in health, particularly compared to individuals in receipt of traditional methods of care delivery. Finally, understanding staffs' perspectives of working in this way would be valuable to explore.
